# Human papillomavirus (HPV) vaccine effectiveness against anal HPV-16 and HPV-18 infections among young men who have sex with men visiting the sexual health center of Amsterdam (HPV4M): study protocol of an observational study

**DOI:** 10.1136/bmjopen-2025-101634

**Published:** 2025-08-26

**Authors:** Sarah van Veelen, Catharina Alberts, Elske Hoornenborg, Sylvia M Bruisten, Koenraad Vermeij, Fiona van der Klis, Luann Noordpool, Johannes A Bogaards, Maarten Franciscus Schim van der Loeff

**Affiliations:** 1 Department of Infectious Diseases, Public Health Service (GGD) of Amsterdam, Amsterdam, The Netherlands; 2 Amsterdam Institute for Immunology & Infectious Diseases (AII), Amsterdam UMC Locatie AMC, Amsterdam, The Netherlands; 3 Amsterdam Public Health Institute (APH), Amsterdam UMC Locatie AMC, Amsterdam, The Netherlands; 4 Department of Epidemiology and Data Science, Amsterdam UMC - Locatie AMC, Amsterdam, The Netherlands; 5 Public Health Laboratory, Public Health Service (GGD) of Amsterdam, Amsterdam, The Netherlands; 6 Soa Aids Netherlands, Amsterdam, The Netherlands; 7 Laboratory for Immunology, National Institute for Public Health and the Environment (RIVM), Bilthoven, the Netherlands

**Keywords:** Anus Neoplasms, Sexual and Gender Minorities, HPV Vaccine, HPV Infection, Public health, Human Papillomavirus Viruses

## Abstract

**Introduction:**

Men who have sex with men (MSM) are at increased risk for human papillomavirus (HPV)-related anal cancer and may benefit from targeted vaccination campaigns, but it is unclear whether vaccination of sexually active MSM still prevents anal HPV infections. Protocolised data on vaccine effectiveness (VE) in routine vaccination programmes targeting MSM are limited. We describe a protocol to evaluate the VE against anal HPV-16/18 infections in young MSM.

**Methods and analysis:**

This observational study compares HPV-16/18 infection prevalence between vaccinated and unvaccinated MSM of the same age. We recruit MSM at the Sexual Health Center of Amsterdam, born in 1996–2003, who receive bivalent HPV vaccination (Cervarix) (group 1). Participants complete a questionnaire on demographics and sexual behaviour and provide anal, penile, oral gargle and venous blood samples at months 0 and 24. Adverse events are monitored by a questionnaire 2 weeks post vaccine. Recruitment of group 2, also MSM born in 1996–2003, but not vaccinated against HPV, will start 24 months after recruitment of group 1. The same samples are collected, and the same questionnaire is administered. Anal samples are tested for HPV DNA and, if positive, typed for 25 HPV genotypes including HPV-16/18; serum samples are tested for HPV-16/18 antibodies; other samples are stored for future analyses. Type-specific VE will be estimated as 1–OR, where OR refers to the OR of DNA positivity in group 1 at month 24 versus DNA positivity of group 2 for the respective vaccine types. Recruitment of group 1 started in February 2023 and was completed in June 2024. From February 2025 onwards, group 1 will return for the 24-month visit and recruitment of group 2 will start.

**Ethics and dissemination:**

This study has been approved by the medical ethics committee of the Amsterdam University Medical Center. This protocol presents minimal risk to participants. Findings will be presented to stakeholders in immunisation policy and be disseminated through scientific journals and conference presentations.

**Trial registration number:**

**Clinical Trial Information System 2022-502224-49-00.:**

**STRENGTHS AND LIMITATIONS OF THIS STUDY**

- Population-level vaccine effectiveness (VE) will be estimated based on a comparison of the human papillomavirus (HPV) prevalence between two comparable groups, using a 2-year buffer period between vaccination delivery at the study clinic and infection status assessment.
- The study is powered to detect VE against newly acquired anal infections in the presence of persistent infections, for which HPV vaccination is ineffective.
- Results of this study will provide valuable insights into whether vaccinating sexually experienced men who have sex with men will be beneficial.
- This study, being observational rather than randomised, may be subject to confounding and VE estimates may be affected despite model-based corrections.
- The study does not allow estimation of VE regarding persistent anal HPV infections or regarding anal disease.

## Introduction

 Anal cancer incidence and mortality have increased globally over the last three decades among men and women.^1^ The highest incidence of anal cancer is found among men who have sex with men (MSM), with an estimated incidence rate of 19 per 100 000 person-years among HIV-negative MSM and 85 per 100 000 among MSM with HIV.^2 3^ Anal cancer is a serious condition with debilitating treatment sequelae and a 5-year survival ranging from 36% to 83% depending on the stage at diagnosis.^4^ Persistent infection with sexually transmitted high-risk anal human papillomavirus (HPV) is the major cause of anal cancer. Whereas HPV-16 and HPV-18 are known as the most common causative types in cervical cancer, causing ~77% of all cervical cancers,^5^ HPV-16 is by far the most carcinogenic type for anal cancer; 90% of anal cancers are caused by HPV-16.^2 6 7^ In a systematic review, the pooled prevalence of anal HPV-16 infections in individuals over 16 years old was 14% in HIV-negative MSM and 29% among MSM with HIV,^8^ compared with 1.8% among HIV-negative men who have sex with women (MSW)^8^ and 4% in women. Although most anal HPV infections clear within 2 years, clearance rates are lower in MSM when compared with MSW and women, increasing their risk for HPV-related anal cancer.^9 10^

Although some HIV guidelines recommend anal cancer screening for people living with HIV, particularly for MSM with HIV, there is no international consensus on routine anal cancer screening.^11^,^15^ HPV vaccination is seen as the best option for the prevention of anal cancer. Licensed prophylactic HPV vaccines include bivalent, quadrivalent and non-avalent vaccines. All HPV vaccines have been shown to be safe and immunogenic in both men and women.^16^,^19^ Although all vaccines target HPV-16 and HPV-18, the vaccines differ in the number of other HPV types included in the adjuvant used and in cross-protective effectiveness against HPV types not included in the vaccine.^20^ The quadrivalent and non-avalent vaccines also include HPV-6 and HPV-11, which cause the majority of anogenital warts and recurrent respiratory papillomatosis. Non-avalent vaccines additionally target five other oncogenic HPV types (HPV-31, 33, 45–52 and 58), predominantly found in cervical disease. Current HPV vaccines have no therapeutic effect, and therefore, effectiveness of HPV vaccination is highest when given at a young age, that is, before sexual exposure to HPV.

Although most effective when given prior to HPV exposure, HPV vaccination can still be effective when administered at later ages. The per protocol analysis of a vaccine efficacy trial among young MSM with no prior HPV exposure reported 94% efficacy against anal HPV-16 DNA detection at any time and 100% for HPV-18.^21^ Effectiveness among young MSM (ages 16–30 years) with prior HPV exposure is much lower, ranging from 45% to 70% for HPV-16^21 22^ and from 45% to 52% for HPV-18.^23 24^ One study even found a non-significant negative effectiveness against HPV-16 among MSM vaccinated between 18 years and 26 years.^24^ Several studies focused on vaccine effectiveness (VE) for all quadrivalent vaccine-targeted types combined, rather than separately assessing VE for HPV-16 and for HPV-18. Reported effectiveness ranged from 20% to 76% for grouped HPV-6/11/16/18,^2122 24^,^26^ with one study demonstrating no significant effectiveness.^23^ These heterogeneous results may be attributed to differences in study design, such as limiting inclusion criteria to participants with few sexual partners,^21^ excluding HPV-positive individuals at baseline,^21^ relying on self-reported HPV vaccination status^22^,^26^ or including people who had been vaccinated very recently among the vaccinated population.^22 26^ These aspects limit insights into the real-world VE as they can lead to overestimations or underestimations of the effectiveness in populations with prior exposure to HPV. Most studies on VE among MSM concerned the quadrivalent vaccine, few concerned the non-avalent vaccine^24^ and none the bivalent vaccine. A Dutch observational study assessed the impact of bivalent HPV vaccination at 12–16 years of age on anal HPV infections in women aged 18–26 years old, showing a 90% (95% CI: 63% to 97%) reduction in the risk of anal vaccine type HPV infections among vaccinated women compared with unvaccinated controls.^27^

In the Netherlands, regional Public Health Services operate Sexual Health Centres (SHC), where certain key populations can get tested and treated for sexually transmitted infections (STIs) free of charge. These clinics also offer hepatitis B vaccination for target groups, such as MSM. SHCs may also be a logistically feasible setting to reach MSM for HPV-related disease prevention, as individuals disclose their sexual orientation, are at increased risk for acquiring HPV, and many may remain susceptible to HPV exposure throughout their lives. Prophylactic HPV vaccination could potentially prevent future HPV infections within this population. However, it is unclear what the effectiveness of the bivalent HPV vaccine among this target group is. Such evidence is crucial for informing public health strategies; if effective against future infections, targeted vaccination of MSM could significantly accelerate the prevention of anal cancer in this population.^28^

In this study, we aim to assess the VE of the bivalent HPV vaccine against anal HPV-16 and HPV-18 infections in MSM aged 19–26 years visiting the SHC of Amsterdam. The focus on HPV-16 and HPV-18 derives from the fact that the vaccine used in this study is designed to prevent infection from HPV types 16 and 18, and because these are the most important oncogenic types with respect to male anogenital and oropharyngeal cancers: about 90% of anal HPV-related cancers,^6 7^ around 70% of penile HPV-related cancers^29^ and about 90% of HPV-related oropharyngeal cancers are caused by HPV-16/18.^30^ MSM have most likely already had multiple sexual partners and been exposed to HPV. Although these factors may reduce VE compared with vaccination at a younger age, we hypothesise that most individuals who have an anal HPV infection at the time of vaccination will clear this infection, and vaccination will protect them against new infections. Therefore, with this study, we aim to elucidate whether HPV vaccination is effective against future infections in a routine public health setting among this sexually active young MSM population.

## Methods and analysis

### Project status

Recruitment for participants of group 1 began on 1 February 2023 during the Dutch national HPV catch-up campaign; inclusion of 431 participants was completed in June 2024. In February 2025, recruitment of group 2 and 24-month visits of group 1 commenced. The results are expected late 2026.

### Objectives

Here we describe the observational study design to estimate the HPV VE for MSM visiting the SHC of Amsterdam (HPV4M study; Clinical Trial Information System registration number 2022-502224-49-00).

The primary aim of the HPV4M study is to evaluate the direct VE of the bivalent HPV vaccine (Cervarix) against anal HPV-16 and HPV-18 DNA positivity among MSM aged 19–26 years, by comparing the prevalence of anal type-specific HPV infections between unvaccinated MSM and vaccinated MSM, 2 years after vaccination.

Our secondary aims are:

1. To assess the anal DNA prevalence, the seroprevalence of HPV-16/18 and the determinants of HPV-16/18 infections.
2. To assess the impact of age and number of lifetime sex partners on VE.
3. To assess the serological response after vaccination and factors affecting serological response.
4. To assess how HPV-16/18 anal positivity 24 months after start of vaccination is affected by HPV DNA and antibody status at the moment of vaccination.
5. To assess the proportion of participants who complete the vaccination schedule and the incidence of adverse events (AE) after administration of Cervarix.
6. Finally, to collect samples and data allowing for later estimations of VE against penile and oral HPV infections.

### Study design

**Figure 1:**
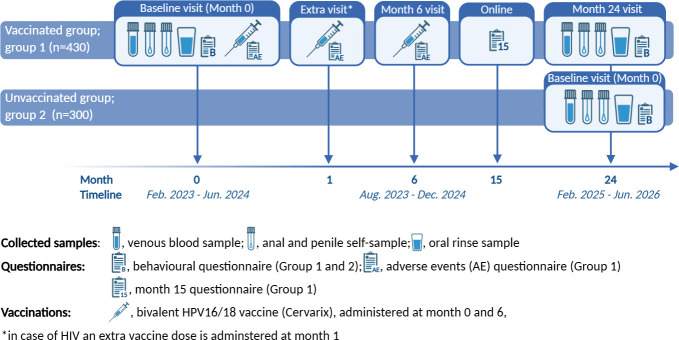
Schematic representation and timeline of HVP4M study. HPV, human papillomavirus. HVP4M, HPV vaccine effectiveness study among MSM.

The HPV4M study is an observational cross-sectional study among young MSM, with a longitudinal component for the vaccinated group (see figure 1 for schematic representation and timeline).

### Study population

In the Netherlands, the bivalent HPV vaccine is routinely offered to preadolescent girls since 2009 and vaccination was extended to boys in 2022. In 2023, a catch-up campaign was launched to offer the bivalent vaccine to all unvaccinated individuals up to the age of 26 years. The vaccination programme uses a 2-dose schedule in accordance with the Dutch national vaccination programme,^31^ that is, vaccine is given at month 0 and at month 6. People with HIV or other causes of immunosuppression are given an extra dose at month 1.

The HPV4M study was initiated during the national catch-up campaign, but was conducted independently. During the study period, HPV vaccination was available at the same clinic through that national catch-up campaign. The age restriction in the HPV4M study is based on the minimum and maximum age within the national catch-up programme.

Participants are recruited at the SHC of Amsterdam in two phases. During the first recruitment phase, MSM born between 1 January 1996 and 31 December 2003 (aged 19–26 years at the start of the catch-up campaign) attending the SHC and accepting HPV vaccination were invited to participate (group 1). MSM were defined as individuals who were assigned male sex at birth, had not undergone genital gender-affirming surgery, regardless of their current gender identity, and who reported having had sex with partners with a penis in the past 6 months. See table 1 for remaining eligibility criteria. Of note, vaccination leads to seroconversion and very high antibody concentrations in the large majority of people with HIV also.^32^,^36^ Therefore, we did not exclude MSM with HIV from participation. For participants with HIV, CD4 cell counts and HIV viral load data are collected. The expected number of MSM with HIV in this age group is low (<5% of eligible men), so stratified analyses by HIV status will not be feasible. In the analysis, we will conduct a sensitivity analysis excluding people with immunocompromising conditions or the use of immunosuppressive medications or HIV infection.

**Table 1:** Overview of eligibility criteria for group one and group 2, HPV4M study, Amsterdam, the Netherlands, 2023–2026

Inclusion criteria	
Group 1	Group 2
Being male and reporting sex with other men in the preceding 6 months Born between 1 January 1996 and 31 December 2003 (age 19–27 years during inclusion) Attending the SHC in Amsterdam Accepting prophylactic HPV vaccination (as routinely offered within the Dutch catch-up programme) Willing and able to return for a subsequent visit for vaccination Planning to stay in or around Amsterdam over the next 2 years Willing and able to complete an online questionnaire at month 15 Willing and able to return for a month 24 final study visit Providing informed consent	Being male and reporting sex with other men in the preceding 6 months Born between 1 January 1996 and 31 December 2003 (age 21–29 years during inclusion) Attending the SHC in Amsterdam Hypothetically accepting prophylactic HPV vaccination Providing informed consent
**Exclusion criteria**	
Both group 1 and group 2	
Having been vaccinated with one or more doses of any HPV vaccine Not able to read/understand Dutch or English Previous enrolment in the study History of anal cancer or anal intraepithelial neoplasia (AIN) Unlikely to adhere to the study protocol in the opinion of the study physician Allergy to one or more components of the vaccine*	

* We asked patients whether they had ever experienced an allergic reaction to a vaccine or to specific components of any medication.

HPV, human papillomavirus; HPV4M, HPV vaccine effectiveness study among MSM; MSM, men who have sex with men; SHC, Sexual Health Center.

Group 1 will be followed up until 24 months after their first vaccination. In the calendar period that group 1 men return for their 24-month visits, an unvaccinated age-matched control population will be enrolled (group 2) (see figure 1 for schematic representation). Group 2 participants are recruited from the same birth cohorts as group 1 participants, enabling direct comparison of the prevalence of anal HPV-16 and HPV-18 infections between the vaccinated (group 1) and the unvaccinated (group 2) participants. During the recruitment period for group 1, HPV vaccination was available free of charge through the national catch-up campaign for all individuals up to the age of 26 years. Group 2 is recruited after the campaign was concluded, and free vaccination was no longer available. Group 2 participants were asked about their reasons for not having received the HPV vaccine. Participants were eligible for participation in group 2 of the study if they would be willing to get vaccinated if offered the vaccine at enrolment.

Ideally, the study population is representative of all MSM aged 19–26 years attending the SHC at the Public Health Service of Amsterdam, in terms of sexual orientation (eg, sex with only men, sex with both women and men), ethnicity, education, socioeconomic status, number of sexual partners, HPV infection status, presence of coinfections (eg, HIV) and past STIs. However, enrolled participants may differ from this population, as participation depends on willingness and ability to participate in this study. To address this, we will collect information on the above-mentioned variables for both study groups and compare their distribution with that of the reference population from the SHC of Amsterdam.

### Recruitment

All MSM born between 1996 and 2003, attending the SHC of Amsterdam, receive an HPV4M-leaflet and are invited to participate in the study. A SHC nurse explains the study procedures and what participation entails. At this point, the person can either decline, participate immediately after providing an informed consent or provide contact details to be contacted later if they are interested on further consideration. If the person declines, the regular SHC visit continues and the decision is noted, so he will not be approached again. Recruitment also takes place through other channels, that is, by distribution of flyers at the SHC and other places frequented by the target population and via social media. The flyers include a quick response code that allows potential participants to register online through a website of the SHC.

### Procedures and interventions

When a potential participant (either group 1 or 2) meets with a research nurse, the study is explained and eligibility criteria are assessed. If he decides to participate, both the participant and research nurse sign two copies of an informed consent form, which includes a Patient Information Form; one copy is kept for our records and the other is given to the participant. Since Group 1 is followed up for 24 months, and participation in Group 2 only consists of a single visit, both procedures are explained separately below.

### Group 1

During the first visit (month 0), participants are asked to complete a short online questionnaire on an SHC-provided tablet (further explained in the Questionnaires section). Next, the nurse explains the sampling procedures, and the participant provides anal, penile and oral rinse self-samples. For the anal swab, participants insert the swab 3 cm into the anal canal and turn it around for 5–10 s. For the penile swab, participants rub the swab firmly over the skin of the penile shaft, including the outside of the foreskin, for 20 s (ie, 5 s per side). For the oral rinse samples, participants rinse their mouth with a saline solution for 30 s, collecting cells and viral particles from the oral cavity and oropharynx. Finally, the nurse collects 5 mL venous blood. If needed, the visit is combined with a regular STI visit (eg, PrEP consultation, STI tests or treatment). After sample collection, the first dose of the bivalent HPV vaccine, Cervarix, is administered. The next appointment (at month 6 and, if necessary, at month 1) is scheduled during this appointment.

At month 6, participants receive their last vaccine dose. Participants who missed a scheduled dose are contacted by phone and mail to reschedule the appointment as soon as possible. Samples for testing are collected neither at month 1 (for participants with HIV) nor at month 6. At month 15, participants complete a short online questionnaire about sexual behaviour and AEs. During the 24 month visit, participants are asked to complete the same online questionnaire and provide the same anal, penile, oral and blood samples as during the initial appointment. Participants who have missed their 6-month (or 1-month dose in case of immunocompromised individuals and people with HIV) vaccine dose are offered vaccination during the 24-month study visit. Study participation is completed after this visit. When all study visits are completed, participants interested in their serological response and anal HPV status will receive their results by mail, along with careful interpretation.

### Group 2

Group 2 will be recruited in the same calendar period when group 1 participants return for their 24-month visit. Men from group 2 will provide the same (anal, penile, oral gargle, venous blood) samples for HPV testing and complete the same questionnaire as group 1. Participation in group 2 consists of one single study visit.

### Adverse events

AEs are monitored through an online questionnaire, sent 2 weeks after administration of each vaccine dose (see the Questionnaires section below). Completed questionnaires are checked on a daily basis to identify any AEs that may be serious (SAEs). In the case of possible SAEs, the participant is contacted by telephone to obtain more details, and in some cases, asked to visit the clinic for further examination. SAEs will be reported to the sponsor (ie, Public Health Service (GGD) of Amsterdam) within 24 hours of obtaining knowledge of the events. The sponsor will report SAEs within 7 days to euclinicaltrials.eu, in accordance with international guidance. Symptoms that are severe will be followed up by the study physician with the assistance of the study nurse, until resolution or stability of the symptoms.

Information on AEs after vaccine administrations at 0 months, 1 month and 6 months (group 1) will be analysed as an additional study outcome. We will provide descriptive statistics of AEs.

### Data collection

#### Clinical data

We will obtain clinical data of all participants using both the medical records collected at the SHC and additional online questionnaires. The following data are collected: (1) demographics (age, gender at birth, highest educational level, work status, income, country of birth); (2) general health (including HIV status, STI history, use of alcohol, any drugs and tobacco), (3) sexual behaviour (age of sexual debut; age of anal sex debut; gender of sex partners; number of sex partners in previous 6 months; number of lifetime sex partners; number of anonymous sex partners in previous 6 months; position during anal sex (ie, insertive/receptive), condom use during anal sex).

#### Questionnaires

During the enrolment visit, participants of both groups are asked to complete a short baseline questionnaire on an SHC-provided tablet concerning above-mentioned topics. In addition, group 2 will be asked the reason for not yet being vaccinated against HPV and whether, if they were to be offered HPV vaccination at that visit, they would accept this offer. Furthermore, group 1 is asked to complete four additional (for a total of five) questionnaires (see figure 1 for overview). Participants of group 1 are asked to complete a brief online questionnaire 2 weeks after each vaccine dose, eliciting information regarding AEs. At month 15, participants of group 1 are asked to complete an additional online questionnaire on sexual behaviour and on AEs. All online questionnaires are sent by email. Participants who do not complete an online questionnaire within 7 days are sent a reminder with a new link. When participants come for a second or later study visit and they have not yet completed a previous online questionnaire, they are asked to complete the AE questionnaire at that visit. At the final visit for participants of group 1, a 24-month questionnaire will be administered with similar questions as during the enrolment visit (see online supplemental appendix I for all questionnaires).

#### Laboratory analyses

Anal, penile, oral gargle and venous blood samples are collected. Penile and oral gargles are stored for future analyses. Anal samples will be tested for HPV DNA at the Public Health Laboratory of the Public Health Service of Amsterdam. First, DNA will be extracted.^37^ Next, a broad-spectrum HPV DNA amplification will be performed using the HPV SPF10 DEIA/line probe assay (LiPA)25 system (Cerba Research; Paris; France). If HPV DNA is detected by the DEIA, it will be genotyped for 25 HPV genotypes (HPV-6, 11, 16, 18, 31, 33, 34, 35, 39, 40, 42, 43, 44, 45, 51, 52, 53, 54, 56, 58, 59, 66, 68/73/97, 70 and 74) using the LiPA.^38^ Positive hybridisation is exposed as purple bands on a blot, which will be independently interpreted by two readers. In case of discrepancies, a third reader will be decisive.^38^

Serum samples will be analysed for HPV-specific Immunoglobin G serum antibodies against L1 virus-like particles (VLP) of HPV types included in the vaccine. The analysis will be done using a VLP-based multiplex immunoassay at the Centre for Infectious Disease Control of the Dutch National Institute for Public Health and the Environment (RIVM).

### Sample size calculation

The sample size was calculated under the assumption that HPV vaccination of MSM aged 19–26 years will have similar efficacy against newly acquired HPV-16 and HPV-18 infections as among MSM with limited prior exposure to these types. We estimated the expected population-level VE in an MSM population attending the SHC in Amsterdam using assumed ‘true’ prophylactic vaccine efficacy from an existing trial^21^ and population-level characteristics of the MSM population at the SHC. Next, we determined the required sample size to demonstrate this expected VE at a significance level of 0.05 with 80% power.

For efficacy against newly acquired infections, we used per-protocol (PP) estimates of the quadrivalent HPV vaccine trial in US men.^21^ The PP population consisted of subjects who were seronegative on day 1 and PCR-negative throughout the 3-dose vaccine course for relevant HPV types. In PP analyses, a prophylactic efficacy of 84.5% (95% CI 63.1% to 94.6%) against anal detection of DNA of HPV-16 and/or HPV-18 at any time was estimated,^21^ which we took as efficacy against incident HPV-16 and HPV-18 DNA positivity in anal samples for our study. The sample size calculation was further informed by prevalence of anal HPV-16 and HPV-18 infections and seropositivity data from MSM participants in the PASSYON study. PASSYON is a biennial repeated (since 2009) cross-sectional study among SHC attendees aged 16–24 years, enrolled at nine SHCs throughout the Netherlands, among which was the SHC of Amsterdam (online supplemental table S1).^39 40^ Note that prevalence and seropositivity were higher in the PASSYON study than in the quadrivalent vaccine trial, reflecting a higher prior exposure to HPV. This is in line with the fact that >80% of the PASSYON participants reported >5 sex partners, whereas the preselected population in the quadrivalent vaccine trial was limited to men with ≤5 lifetime sex partners.

**Figure 2:**
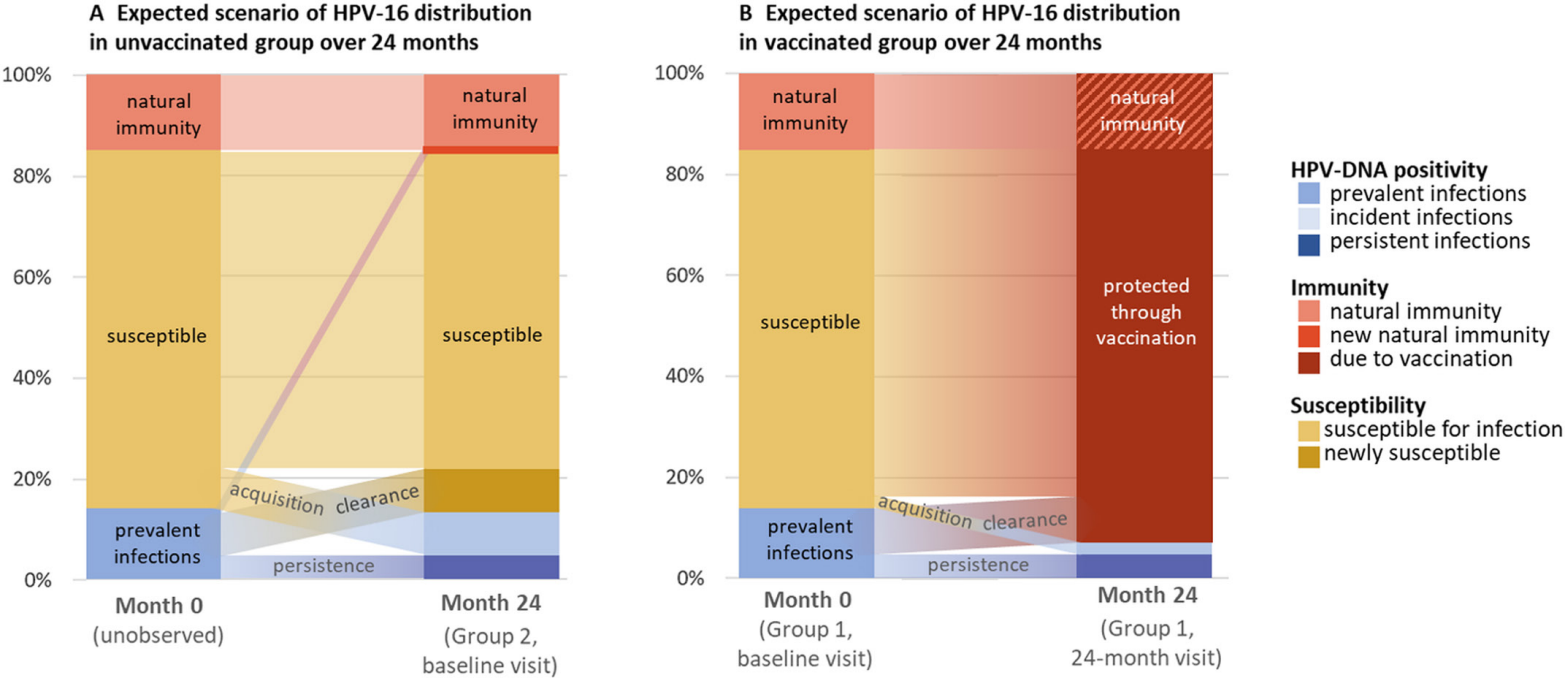
Expected HPV-16 population distribution among young MSM in a vaccinated and unvaccinated scenario. HPV, human papillomavirus; MSM, men who have sex with men.

In sample size calculation, we made two assumptions. First, we assumed that anyone who tests positive for a vaccine type (either DNA-positive or seropositive) at baseline is not at risk for acquisition of that vaccine type during follow-up and second, that there are no latent or undetectable (DNA-negative) HPV infections at baseline (see figure 2). By powering our study to a scenario without latency, we aim to detect VE that is relevant for achieving substantial population-level impact in a targeted immunisation campaign. We previously concluded, based on a dynamic modelling study, that the efficiency of targeted vaccination would be severely reduced if latency were common among sexually experienced vaccine recipients.^28^ We deliberately did not power our study to capture diminished VE in such a scenario.

Following these assumptions, we assume a baseline prevalence of 13.9% for HPV-16 and 11.3% for HPV-18 in both groups and calculated an expected 24-month DNA positivity of 6.2% for HPV-16 and 4.6% for HPV-18 in group 1. By using the χ^2^ test to compare the 24-month prevalence in group 1 against the baseline prevalence in group 2, a sample size of 264 for HPV-16 and 284 for HPV-18 is required for 80% statistical power. This corresponds with an OR of 0.41 for HPV-16 and 0.38 for HPV-18*,* which leads to an anticipated VE against anal HPV-16 and HPV-18 of 59% and 62%, respectively (see online supplemental table S1, figure S1 and appendix II for formula and calculations). The sample size was determined at 300 vaccinated and 300 unvaccinated MSM, to retain power when estimating VE in analyses with adjustment for potential confounders. In order to have complete data for 300 vaccinated men at month 24, with an expected loss to follow-up (LTFU) of 30% among group 1 participants, the target for inclusion was set at 430 participants in group 1. The 30% expected LTFU over 24 months is based on a similar previous study among MSM visiting the SHC, where 20% LTFU was observed.^41^

### Statistical analyses

#### Primary outcomes

Our main endpoint is type-specific VE against anal vaccine type infection, derived from the comparison of HPV-16 or HPV-18 DNA positivity in anal samples between group 1 participants at 24 months after vaccination and unvaccinated participants of group 2. VE will be estimated as 1–OR, where OR stands for the OR of HPV-16 DNA positivity in group 1 at 24 months relative to the HPV-16 DNA positivity of group 2. The use of ORs to estimate VE is suitable in this cross-sectional setting because it captures the joint effects of vaccination on the infection and clearance hazards,^42 43^ and our study is built on the premise that MSM may experience repeated events of HPV acquisition and clearance at a type-specific level. Prophylactic vaccination is anticipated to lead to reduced acquisition of new vaccine-type HPV infections, and our study is powered to detect a difference in vaccine-type DNA positivity due to this reduction.

Since our study is not randomised, the comparison between the two study groups and VE estimates may be affected by confounding factors. To address this, we will compare group 1 at month 24 and group 2 to identify possible differences between the groups that may lead to biased estimates. Groups will be compared using standard statistical tests. Continuous variables will be analysed using unpaired t-tests in case of normally distributed data, and Mann-Whitney’s U-tests in case of skewed data. For categorical variables, we will use χ^2^ tests or Fisher’s exact test in case of expected cell count less than five. Statistical significance will be considered at a significance level of 0.05 without correction for multiple testing. Variables with well-known effect on HPV risk (such as number of sex partners), and variables with significantly different distributions between both groups will be included in a regression model to obtain adjusted estimates of VE.

We will calculate adjusted ORs for HPV-16 and HPV-18 positivity using a logistic mixed model, adjusting for known variables that are associated with HPV positivity (all types) or vaccination status. This method has been used before to estimate type-specific VE of the bivalent HPV vaccine against cervicovaginal and anal HPV positivity in female SHC clients, with high concordance between both estimates.^27 44^

We will provide both intention-to-treat (ITT) and PP estimates of VE. In the ITT analysis, all individuals who are recruited into group 1 and return for the month 24 visit will be included. In the PP analysis, we will only consider group 1 participants who received all vaccine doses in the recommended intervals. Recommended intervals are 5–12 months after the first dose for the 6-month dose, and 3–6 weeks after dose 1 for the 1-month dose in immunocompromised individuals.^45 46^ In a sensitivity analysis, we will also calculate VE among the subgroup of PP participants with confirmed antibody response at month 24. Our primary analyses will be based on complete data for HPV-16/18 infection outcomes, using model-based imputation to handle any missing data for possible confounders (ie, covariates). In a sensitivity analysis, we will use model-based imputation for missing HPV-16/18 infection outcomes, to assess the effect of potential selective drop-out in Group 1 participants from baseline to month 24.

#### Secondary outcomes

For all vaccinated participants, we will collect information on 2-dose completion according to the 0-month and 6-month schedule for HIV-negative MSM and the 0-month, 1-month and 6-month schedule for participants with HIV. Vaccination completion will be reported as proportions with 95% CIs from a binomial distribution. Using logistic regression analysis, we will assess determinants of completing the vaccination schedule among those who received a first HPV vaccination.

We will assess the proportions of HPV-16 and HPV-18 anal positive men and assess the proportions that are both HPV DNA-negative and seronegative for HPV-16 and for HPV-18 at baseline, both in group 1 and in group 2. This proportion gives an indication of the proportion of men at immediate risk of vaccine-type infection and hence to achieve direct benefit from vaccination. Using logistic regression analysis, we will assess determinants of anal HPV-16/18 infection and also of combined anal DNA-positivity and seropositivity.

In group 1, we will assess the proportions who have seroconverted, separately for each HPV type included in the vaccine. We will assess the geometric mean concentration (GMC) of type-specific antibodies and assess the impact of various baseline variables (like age, number of lifetime sexual partners, report of anal sex, Ab status at baseline, HPV DNA status at baseline) on the GMC, using multivariable linear regression analysis of (if needed log-transformed) HPV-16 and HPV-18 antibody concentrations separately.

For group 1 participants, we will assess how HPV-16/18 positivity 24 months after start of vaccination is affected by their HPV DNA and HPV antibody status just prior to vaccination. This will be done by calculating type-specific positivity rates, stratified by baseline DNA and antibody status and by estimating the effect of both homologous and heterologous DNA or antibody positivity at baseline on type-specific positivity rates at 24 months. We previously estimated a 24-month clearance probability of 65% for prevalent HPV-16 infections in HIV-negative MSM in Amsterdam, with a median age of 38.^28 41^ These analyses could shed light on the effectiveness of prophylactic vaccination in relation to prevalent infection or prior exposure to HPV at the moment of vaccination.

### Patient and public involvement

Patient and/or public were involved in the design of this research. Representatives were selected from the target community to contribute ideas and provide input on the design and recruitment of participants, ensuring that the perspectives and interests of the target population were considered.

### Ethics and dissemination

This study has been reviewed and approved by the Medical Ethics Committee of the Amsterdam University Medical Center and registered accordingly in the European Clinical Trials Information System (2022-502224-49-00).

Data collected in this study are pseudonymised at the point of entry (inclusion visit) and stored on a secured folder with access restricted to the principal investigator and research personnel. After data collection, data from the questionnaires, SHC of Amsterdam and laboratory are merged based on unique study numbers. Data are checked for completeness and accuracy, and all analyses are version-controlled. At the end of the study, anonymised datasets and analysis code will be archived for future reference and may be made available to other researchers on reasonable request and ethics approval. The data will be stored for 25 years and the laboratory samples for 10 years.

The study protocol is made public through this protocol paper. Findings from this study will be published in peer-reviewed journals and presented at both national and international conferences. The study design was presented as a poster at the International Papillomavirus Conference in Edinburgh in November 2024. In terms of knowledge translation, the empirical evidence obtained with this study could provide insights for policymakers in the Netherlands and elsewhere. Therefore, results will be presented to stakeholders in immunisation policy and be shared through media aimed at a broad audience.

## Discussion

Targeted vaccination programmes for MSM could provide substantial health benefits, as they face high risk of HPV infections and related diseases, particularly anal cancer.^2 3 9 10^ The efficacy of vaccinating MSM up to 26 years of age with limited prior exposure to HPV is high,^21^ but estimates of real-world effectiveness vary, likely due to variations in study design, in particular regarding prior HPV exposure among study participants.^22^,^2647^ Previous studies lacked prevaccination HPV DNA data and antibodies data and often had to rely on self-reported vaccination status or included individuals who were very recently vaccinated; all these factors impact VE estimates. Furthermore, VE estimation in HPV-exposed populations is highly dependent on the proportion of prevalent infections, the clearance rate, natural immunity and ongoing HPV exposure. The HPV4M study addresses these knowledge gaps by evaluating the effectiveness of the bivalent HPV vaccine at population level among young MSM visiting the SHC of Amsterdam. Our findings can answer the question of whether targeted HPV vaccination programmes for MSM up to age 26 years are effective, in order to improve control of HPV-related cancer in MSM.

This study has several strengths. First, the cross-sectional design with a longitudinal component allows efficient VE assessment. The longitudinal component for vaccinated participants provides valuable prevaccination data, minimises bias from self-reported vaccination status and enables us to define the exact time between vaccination and HPV sampling. Furthermore, it is crucial to allow an appropriate clearance period to prevent underestimation of VE due to prevalent infections at the time of immunisation. As such, the 24-month follow-up in this study is based on the 24-month clearance probability of 65% in a previous Dutch study among older HIV-negative MSM (median age: 38 years, IQR 34–42).^38^ More recent studies among younger HIV-negative MSM have estimated 24-month clearance probabilities between 70% and 80%.^9 10^ Our assumption on clearance may therefore be conservative, but prevents us from underestimating the VE. Higher clearance than anticipated will only improve the statistical power of our study. Second, our extensive data collection allows us to correct for factors impacting VE and to identify potential confounders. A randomised controlled trial would have been the best approach to address the research question. However, randomisation was deemed unethical, as the study was integrated into an existing national HPV vaccination catch-up campaign and withholding vaccination would have been unfair to participants willing to get vaccinated. By recruiting all participants at the same SHC using the same approach and protocol, we increase comparability between the vaccinated and unvaccinated groups in the absence of randomisation. We anticipated that recruiting an unvaccinated control group during the campaign could introduce confounding, as those who choose to get vaccinated may differ systematically from those who do not, for example, in terms of health literacy and awareness of HPV-related risks. To address this, the unvaccinated group will be recruited at a later stage (when the HPV catch-up campaign is concluded) and will consist of men who missed the opportunity for HPV vaccination rather than those who explicitly declined it. Attendees were eligible for participation in group 2 of the study if they would be willing to get vaccinated if offered the vaccine at enrolment. Furthermore, the analysis will adjust for variables on which the two groups differ. Nevertheless, residual confounding may occur.

When assessing the sample size, we assumed that individuals with vaccine-type antibodies due to prior infections are not susceptible to new vaccine type infections. Some studies have suggested that naturally acquired antibodies after HPV infection reduce the risk of new infections among women.^48^ However, studies also found that the evidence of natural immunity among men was inconclusive^49^ or suggested lack of protection at naturally occurring antibody levels.^50^ It remains uncertain how long natural immunity lasts, if present at all in men, and what the impact of natural immunity is on vaccine-acquired immunity.^51^ We acknowledge that our assumption may be a simplification of the natural immunity. However, we expect that less immunity is likely to improve the statistical power of our study.

Two key challenges of this study are the potential LTFU among the vaccinated MSM (group 1) and the recruitment of a comparable control group. Regarding the latter, we will use age-matching on group level and recruitment in the same setting and time period to ensure a comparable age distribution and recent population exposure between the vaccinated and unvaccinated groups. Furthermore, we will test for comparability on variables like number of sexual partners, STI history, years of sexual activity, etc, and correct for possible confounders in multivariable analyses. The LTFU of group 1 will be minimised by ensuring personal engagement and attention with the participant, combining the follow-up visit with a regular STI visit and sending regular reminders if needed. Furthermore, our sample size of 430 for group 1 accounts for 30% LTFU, which is higher than observed in a similar study at the SHC of Amsterdam.^38^

The HPV4M study will evaluate whether vaccination programmes targeting MSM aged 19–26 years can effectively prevent newly acquired anal HPV-16/18 infections and hence HPV-related anal cancer. The results of our study can be used to project the population-level impact of HPV vaccination programmes, perform cost-effectiveness analyses of scaled-up HPV vaccination efforts and to inform policymakers in a timely manner regarding the desirability of campaigns targeting high-risk groups with similar HPV exposure (not necessarily restricted to MSM) in parallel with the National Immunisation Programme. If the project demonstrates that HPV vaccination is effective against new anal HPV infections in sexually active MSM up to 27 years of age, routine vaccination could be easily implemented by adding it to the existing targeted hepatitis B vaccination programme in SHC, which has a similar vaccination schedule.

## Supplementary material

10.1136/bmjopen-2025-101634 online supplemental file 1

## References

[1] Mignozzi S, Santucci C, Malvezzi M (2024). Global trends in anal cancer incidence and mortality. Eur J Cancer Prev.

[2] Clifford GM, Georges D, Shiels MS (2021). A meta-analysis of anal cancer incidence by risk group: Toward a unified anal cancer risk scale. Int J Cancer.

[3] Machalek DA, Poynten M, Jin F (2012). Anal human papillomavirus infection and associated neoplastic lesions in men who have sex with men: a systematic review and meta-analysis. Lancet Oncol.

[4] Cancer.Net (2023). Statistics adapted from the american cancer society’s (acs) publication the acs website; the international agency for research on cancer website; and the national cancer institute’s surveillance. https://www.cancer.net/cancer-types/anal-cancer/statistics#:~:text=If%20the%20cancer%20is%20diagnosed,survival%20rate%20is%20about%2067%25.

[5] Wei F, Georges D, Man I (2024). Causal attribution of human papillomavirus genotypes to invasive cervical cancer worldwide: a systematic analysis of the global literature. The Lancet.

[6] Lin C, Franceschi S, Clifford GM (2018). Human papillomavirus types from infection to cancer in the anus, according to sex and HIV status: a systematic review and meta-analysis. Lancet Infect Dis.

[7] Alemany L, Saunier M, Alvarado-Cabrero I (2015). Human papillomavirus DNA prevalence and type distribution in anal carcinomas worldwide. Int J Cancer.

[8] Wei F, Gaisa MM, D’Souza G (2021). Epidemiology of anal human papillomavirus infection and high-grade squamous intraepithelial lesions in 29 900 men according to HIV status, sexuality, and age: a collaborative pooled analysis of 64 studies. Lancet HIV.

[9] Wei F, Goodman MT, Xia N (2023). Incidence and Clearance of Anal Human Papillomavirus Infection in 16 164 Individuals, According to Human Immunodeficiency Virus Status, Sex, and Male Sexuality: An International Pooled Analysis of 34 Longitudinal Studies. Clin Infect Dis.

[10] Tian T, Fu L, Wang B (2024). Clearance of anal and penile HPV 6, 11, 16, and 18 DNA and antibodies among adolescent men who have sex with men (HYPER): An observational cohort study. Vaccine X.

[11] European AIDS Clinical Society (2023). European aids clinical society. https://www.eacsociety.org/guidelines/eacs-guidelines.

[12] National Institutes of Health (2023). Clinical guidelines for the prevention and treatment of opportunistic infections in adults and adolescents with hiv: human papillomavirus disease. https://clinicalinfo.hiv.gov/en/guidelines/hiv-clinical-guidelines-adult-and-adolescent-opportunistic-infections/human?view=brief.

[13] Nederlandse vereniging van HIV behandelaren (NVHB) (2023). Screening for anal premalignant lesions [in dutch]. https://richtlijnhiv.nvhb.nl/index.php/Screening_anale_premaligne_afwijkingen.

[14] Bower M, Palfreeman A, Alfa-Wali M (2014). British HIV Association guidelines for HIV-associated malignancies 2014. HIV Med.

[15] Ong J, McCloskey J, Tong W (2019). HIV management guidelines: human papillomavirus infection and associated malignancies in hiv-infected people: australian society of hiv medicine (ashm). https://hiv.guidelines.org.au/management/human-papillomavirus-infection-and-associated-malignancies-in-hiv-infected-people/anal-cancer/.

[16] Einstein MH, Takacs P, Chatterjee A (2014). Comparison of long-term immunogenicity and safety of human papillomavirus (HPV)-16/18 AS04-adjuvanted vaccine and HPV-6/11/16/18 vaccine in healthy women aged 18-45 years: End-of-study analysis of a Phase III randomized trial. Hum Vaccin Immunother.

[17] Schiller JT, Castellsagué X, Garland SM (2012). A Review of Clinical Trials of Human Papillomavirus Prophylactic Vaccines. Vaccine (Auckl).

[18] Arbyn M, Xu L (2018). Efficacy and safety of prophylactic HPV vaccines. A Cochrane review of randomized trials. Expert Rev Vaccines.

[19] Harder T, Wichmann O, Klug SJ (2018). Efficacy, effectiveness and safety of vaccination against human papillomavirus in males: a systematic review. BMC Med.

[20] (2024). Human papillomavirus vaccines: cervarix, gardasil, gardasil 9, silgard. European Medicines Agency (EMA).

[21] Palefsky JM, Giuliano AR, Goldstone S (2011). HPV vaccine against anal HPV infection and anal intraepithelial neoplasia. N Engl J Med.

[22] Chow EPF, Tabrizi SN, Fairley CK (2021). Prevalence of human papillomavirus in young men who have sex with men after the implementation of gender-neutral HPV vaccination: a repeated cross-sectional study. Lancet Infect Dis.

[23] Chambers C, Deeks SL, Sutradhar R (2023). Vaccine Effectiveness Against 12-Month Incident and Persistent Anal Human Papillomavirus Infection Among Gay, Bisexual, and Other Men Who Have Sex With Men. J Infect Dis.

[24] DeSisto CL, Winer RL, Querec TD (2025). Vaccine Effectiveness Against Anal HPV Among Men Who Have Sex With Men Aged 18-45 Years Attending Sexual Health Clinics in 3 United States Cities, 2018-2023. J Infect Dis.

[25] Chambers C, Deeks SL, Sutradhar R (2022). Anal Human Papillomavirus Prevalence Among Vaccinated and Unvaccinated Gay, Bisexual, and Other Men Who Have Sex With Men in Canada. Sex Transm Dis.

[26] Meites E, Winer RL, Newcomb ME (2020). Vaccine Effectiveness Against Prevalent Anal and Oral Human Papillomavirus Infection Among Men Who Have Sex With Men-United States, 2016-2018. J Infect Dis.

[27] Woestenberg PJ, King AJ, Van Benthem BHB (2020). Bivalent Vaccine Effectiveness Against Anal Human Papillomavirus Positivity Among Female Sexually Transmitted Infection Clinic Visitors in the Netherlands. J Infect Dis.

[28] Bogaards JA, Mooij SH, Xiridou M (2019). Potential effectiveness of prophylactic HPV immunization for men who have sex with men in the Netherlands: A multi-model approach. PLoS Med.

[29] Alemany L, Cubilla A, Halec G (2016). Role of Human Papillomavirus in Penile Carcinomas Worldwide. Eur Urol.

[30] Ndiaye C, Mena M, Alemany L (2014). HPV DNA, E6/E7 mRNA, and p16INK4a detection in head and neck cancers: a systematic review and meta-analysis. Lancet Oncol.

[31] Netherlands HCot (2022). Change to HPV Vaccine Doses The Hague, August 30, 2022 Ed.

[32] Faust H, Toft L, Sehr P (2016). Human Papillomavirus neutralizing and cross-reactive antibodies induced in HIV-positive subjects after vaccination with quadrivalent and bivalent HPV vaccines. Vaccine (Auckl).

[33] Fontes A, Andreoli MA, Villa LL (2016). High specific immune response to a bivalent anti-HPV vaccine in HIV-1-infected men in São Paulo, Brazil. Papillomavirus Res.

[34] Toft L, Storgaard M, Müller M (2014). Comparison of the immunogenicity and reactogenicity of Cervarix and Gardasil human papillomavirus vaccines in HIV-infected adults: a randomized, double-blind clinical trial. J Infect Dis.

[35] Toft L, Tolstrup M, Müller M (2014). Comparison of the immunogenicity of Cervarix® and Gardasil® human papillomavirus vaccines for oncogenic non-vaccine serotypes HPV-31, HPV-33, and HPV-45 in HIV-infected adults. Hum Vaccin Immunother.

[36] Wilkin T, Lee JY, Lensing SY (2010). Safety and immunogenicity of the quadrivalent human papillomavirus vaccine in HIV-1-infected men. J Infect Dis.

[37] Heymans R, van der Helm JJ, de Vries HJC (2010). Clinical value of Treponema pallidum real-time PCR for diagnosis of syphilis. J Clin Microbiol.

[38] Mooij SH, van Santen DK, Geskus RB (2016). The effect of HIV infection on anal and penile human papillomavirus incidence and clearance: a cohort study among MSM. AIDS.

[39] Kusters JMA, Obels I, van der Klis FRM (2024). Prevalence and risk factors for HPV seropositivity and anogenital DNA positivity among men who have sex with men: a repeated cross-sectional study. Int J Infect Dis.

[40] Woestenberg PJ, van Benthem BHB, Bogaards JA (2020). HPV infections among young MSM visiting sexual health centers in the Netherlands: Opportunities for targeted HPV vaccination. Vaccine (Auckl).

[41] van Aar F, Mooij SH, van der Sande MAB (2014). Twelve-month incidence and clearance of oral HPV infection in HIV-negative and HIV-infected men who have sex with men: the H2M cohort study. BMC Infect Dis.

[42] Auranen K, Rinta-Kokko H, Halloran ME (2013). Estimating strain-specific and overall efficacy of polyvalent vaccines against recurrent pathogens from a cross-sectional study. Biometrics.

[43] Man I, Auranen K, Wallinga J (2019). Capturing multiple-type interactions into practical predictors of type replacement following human papillomavirus vaccination. Philos Trans R Soc Lond B Biol Sci.

[44] Woestenberg PJ, King AJ, van Benthem BHB (2018). Bivalent Vaccine Effectiveness Against Type-Specific HPV Positivity: Evidence for Cross-Protection Against Oncogenic Types Among Dutch STI Clinic Visitors. J Infect Dis.

[45] Centers for Disease Control and Prevention (2021). HPV vaccination: recommendations of the acip. https://www.cdc.gov/vaccines/vpd/hpv/hcp/recommendations.html.

[46] Rijksinstituut voor Volksgezondheid en Milieu (2025). Tijdstip van vaccinaties (rijksvaccinatieprogramma) [in dutch]. https://rijksvaccinatieprogramma.nl/professionals/richtlijnen/uitvoering/7-tijdstip-van-vaccinaties.

[47] Wei F, Alberts CJ, Albuquerque A (2023). Impact of Human Papillomavirus Vaccine Against Anal Human Papillomavirus Infection, Anal Intraepithelial Neoplasia, and Recurrence of Anal Intraepithelial Neoplasia: A Systematic Review and Meta-analysis. J Infect Dis.

[48] Yao X, Chen W, Zhao C (2021). Naturally acquired HPV antibodies against subsequent homotypic infection: A large-scale prospective cohort study. Lancet Reg Health West Pac.

[49] Beachler DC, Pinto LA, Kemp TJ (2018). An Examination of HPV16 Natural Immunity in Men Who Have Sex with Men (MSM) in the HPV in Men (HIM) Study. Cancer Epidemiol Biomarkers Prev.

[50] Mooij SH, Landén O, van der Klis FRM (2014). No evidence for a protective effect of naturally induced HPV antibodies on subsequent anogenital HPV infection in HIV-negative and HIV-infected MSM. J Infect.

[51] Matthijsse SM, Hontelez JAC, Naber SK (2015). The estimated impact of natural immunity on the effectiveness of human papillomavirus vaccination. Vaccine (Auckl).

