# Supplementary material for: Human papillomavirus (HPV) vaccine effectiveness against anal HPV-16 and HPV-18 infections among young men who have sex with men visiting the sexual health center of Amsterdam (HPV4M): study protocol of an observational study
Source: BMJ Open. 2025 Aug 26;15(8):e101634. doi: 10.1136/bmjopen-2025-101634 (PMC12410653; doi:10.1136/bmjopen-2025-101634)
Supplement: online supplemental file 1 [file bmjopen-15-8-s001.docx]

**Supplemental Material to:**

**Human papillomavirus (HPV) vaccine effectiveness against anal HPV-16 and HPV-18 infections among young men who have sex with men visiting the Sexual Health Center of Amsterdam (HPV4M): study protocol of an observational study**

*Sarah van Veelen^1,2^, Catharina J Alberts^1,2,4^, Elske Hoornenborg^1^, Sylvia Bruisten^1^, Koenraad Vermeij^5^, Fiona van der Klis^6^, Luann Noordpool^1^, Johannes A Bogaards*^2,3,4^, Maarten F Schim van der Loeff* ^1,2,3^*

**Shared last authors*

**Affiliations**

1. Department of Infectious Diseases, Public Health Service (GGD) of Amsterdam, the Netherlands;
2. Amsterdam Institute for Immunology & Infectious Diseases (AII) Amsterdam UMC, University of Amsterdam, Amsterdam, The Netherlands;
3. Amsterdam Public Health Institute (APH), Amsterdam, the Netherlands;
4. Department of Epidemiology and Data Science, Vrije Universiteit, Amsterdam UMC, the Netherlands;
5. SOA AIDS Nederland, Amsterdam, the Netherlands;
6. Laboratory for Immunology, National Institute for Public Health and the Environment (RIVM), Bilthoven, the Netherlands.

**Contents:**

**Table S1** Sample size calculation and assumptions used)2

**Figure S1** Calculations for anal HPV positivity of Group 1 participants at month 0 and month 243

**Appendix I:** Questionnaires

Additional Baseline Questionnaire at inclusion (month 0) and month 245

Online questionnaire two weeks after vaccination (Group 1)6-7

Online questionnaire sent month 158

**Appendix II:** Sample Size Calculations4

**References** 9

**Table S1** Sample size calculation for the HPV4M study and assumptions used

|  |  | HPV-16 | HPV-18 |
| --- | --- | --- | --- |
| θ | Efficacy against anal HPV-16/-18 DNA detection (at any time)^1^ | 0.845 | 0.845 |
| κ | 24-month clearance probability^2^ | 0.65 | 0.70 |
| π_1_ | Expected prevalence in Group 1 month 0 and Group 2^3^ | 0.139 | 0.113 |
| π_2_ | Projected prevalence in Group 1 month 24 | 0.062 | 0.046 |
| Ψ | Odds Ratio* | 0.409 | 0.378 |
| α | Test significance level | 0.05 | 0.05 |
|  | Power (1-β) | 0.80 | 0.80 |
|  | **Sample Size required per group^#^ (n)** | 264 | 284 |

*Odds Ratio is defined by Group 1 and 2 proportions: ψ = π_2_(1 – π_1_)/(π_1_(1 – π_2_))
 ^#^Two-group continuity-corrected χ^2^ test of equal proportions (i.e. Odds Ratio = 1 under H_0_)

Calculations made using nQuery version 8.5.1 (Statistical Solutions 2019)

**Figure S1** Calculations for anal HPV positivity of Group 1 participants at month 0 and month 24


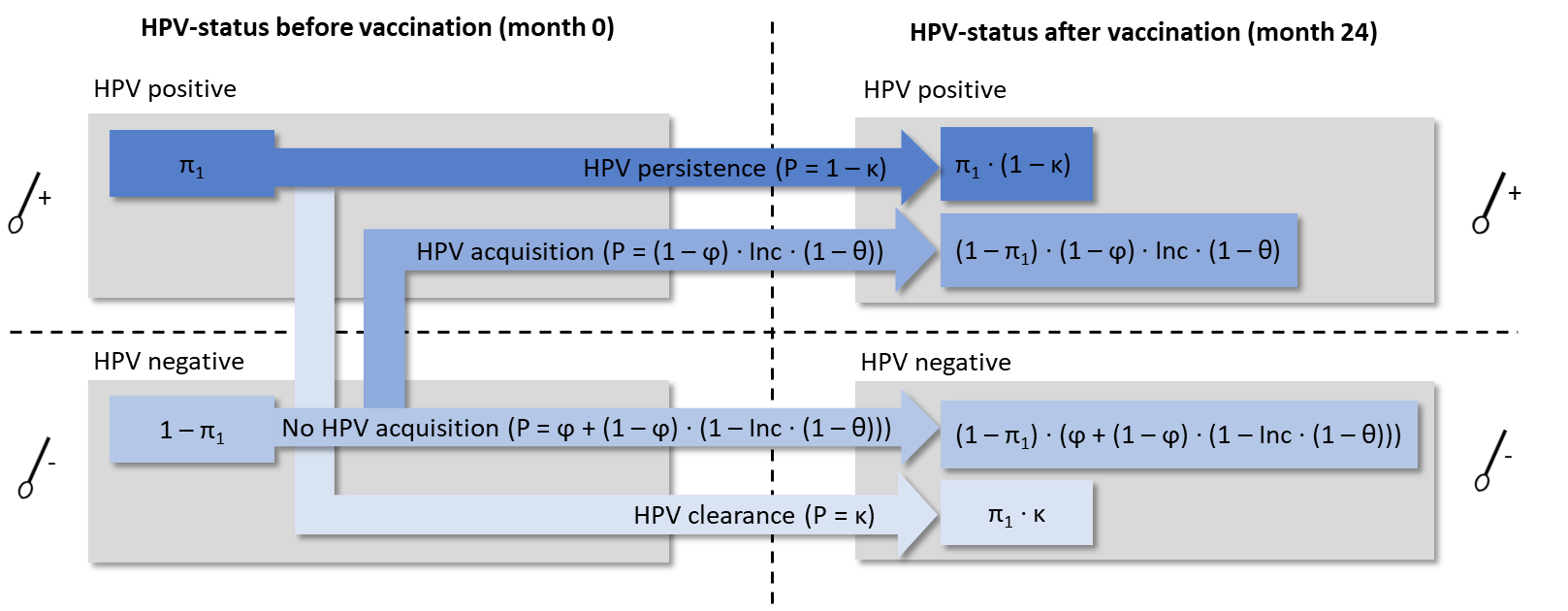


P = probability; κ = 24-month clearance probability; ϕ = proportion immune among HPV-negatives; θ = ‘true’ vaccine efficacy; Inc = per-capita cumulative incidence of infection; π_1_ = expected prevalent infections at baseline

**Appendix I: Questionnaires**

**Behavioral Questionnaire at inclusion (month 0) and month 24**

1. Study number [provided by system]
2. Todays’ date [provided by system]
3. Do you currently have a steady partner? (yes / no)
4. What is your living situation?
   (I live alone / I live with my steady partner / I live with my parents or caretakers / I live with others)
5. Do you currently have paid employment?
   (No, I am studying / No, I work as volunteer / No, I am unemployed / No, I am >80% disabled / No, I am retired / Yes, I work _ _ _ hours per week / Yes, I work _ _ _ hours per week and combine this with studying / Other, …)
6. What is your personal nett monthly income?
   (0-950 euro / 951-1201 euro / 1701-2950 euro / >2950 euro / I prefer not to disclose this)
7. Have you ever used tobacco? (yes/no)
8. If question 7 ‘yes’: Cigarette use
   (current user / ex-user / other specify ( current-user, … / ex-user, …)
9. If question 8 ‘current user’: How many cigarettes? (less than 1 per day / 1-4 per day / 5-10 per day / more than 10 per day)*
10. For how many years have you smoked in total? (years)
11. Have you been circumcised? (yes/no)
12. Have you ever had warts on or near the penis? (yes / no)
13. Have you ever had warts in or around the anus? (yes / no)
14. What is your sexual preference? (your sexual preference does not have to be the same as your sexual behaviour); (exclusively heterosexual / almost entirely heterosexual / mostly heterosexual / equally homosexual and heterosexual / mostly homosexual / almost entirely homosexual / exclusively homosexual)
15. How old were you when you had first sex with a man? (years)
16. How old were you when you had first anal sex with a man? (years)
17. How many different men have you had sex with in your life?
18. With how many partners did you have insertive anal sex in the last 6 months?
19. With how many partners did you have receptive anal sex in the last 6 months?

*Question 9 was added in the 24-month questionnaire for Group 1 and the 0-month questionnaire for Group 2

**Online questionnaire two weeks after vaccination (Group 1)**

| Did you, since you received the vaccine two weeks ago, experience the following signs or symptoms: | Yes/No | If Yes, Please indicate the severity of the symptom*:  mild (1), moderate (2), severe (3) | Start date | Is the symptom still present today?  (yes/no) | End date | Did you visit a physician or a hospital for this symptom? | Remarks |
| --- | --- | --- | --- | --- | --- | --- | --- |
| Pain at injection site |  |  |  |  |  |  |  |
| Swelling at injection site |  |  |  |  |  |  |  |
| Redness at injection site |  |  |  |  |  |  |  |
| Difficulty moving arm in which injection was given |  |  |  |  |  |  |  |
| Skin rash |  |  |  |  |  |  |  |
| Itch |  |  |  |  |  |  |  |
| Fever |  |  |  |  |  |  |  |
| Headache |  |  |  |  |  |  |  |
| Dizziness |  |  |  |  |  |  |  |
| Fatigue |  |  |  |  |  |  |  |
| Nausea |  |  |  |  |  |  |  |
| Vomiting |  |  |  |  |  |  |  |
| Diarrhoea (>3 loose stools per 24 hours) |  |  |  |  |  |  |  |
| Abdominal pain |  |  |  |  |  |  |  |
| Cough |  |  |  |  |  |  |  |
| Aching muscles |  |  |  |  |  |  |  |
| Joint pain |  |  |  |  |  |  |  |
| Upper respiratory tract infection (infection of the nose, throat or trachea) |  |  |  |  |  |  |  |
| General malaise |  |  |  |  |  |  |  |
| Fainting |  |  |  |  |  |  |  |
| Swollen glands (e.g. in the neck, armpit or groin) |  |  |  |  |  |  |  |
| Other, please describe … |  |  |  |  |  |  |  |

***** Mild, moderate and severe are defined as follows: Mild is an event that is easily tolerated, causing minimal discomfort and not interfering with everyday activities; moderate is an event that is sufficiently discomforting to interfere with normal everyday activities; severe is an event that prevents normal everyday activities

1. Have you been admitted to hospital since you received the vaccine two weeks ago? (yes/no)
2. Was this a planned admission or an emergency admission? (planned /emergency)

*(A planned admission is a hospital admission that was scheduled before starting study participation (i.e. before the date of signature of Informed Consent Form).*

1. What was the date of admission? (DD/MM/YYYY)
2. What was the date of discharge? (DD/MM/YYYY)
3. Not discharged yet (yes)
4. What was the reason for the admission?

We may contact you if we have additional questions regarding the above. Thank you!

**Online questionnaire sent month 15**

Dear Sir,

You are receiving this e-mail because you agreed to participate in the HPV4M study, a study investigating how well the HPV vaccine works in men 19-26 years of age.

We want to ask you some additional questions. Could you please complete this questionnaire <<link to questionnaire>>. Completing this questionnaire will take you approximately 5 minutes.

In 9 months we will invite you again for the 2-year visit of this study. We will ask you to provide the same biological samples that you provided during your first visit and complete the same additional questions. If you want, we can offer you a regular consultation.

Thank you for your time!

1. Study number [provided by system]
2. Today’s date [provided by system]
3. Do you currently have a steady partner?
4. How many different people have you had sexual contact with in the last 6 months? The question concerns
   - oral sexual contact (cunnilingus, analingus or blow jobs)
   - penis in vagina
   - penis in the anus
   Each sex partner counts just one time. If you are not exactly sure, you can estimate this.
5. With how many partners did you have insertive anal sex in the last 6 months?
6. With how many partners did you have receptive anal sex in the last 6 months?
7. Have you been diagnosed with HIV in the last 15 months? (yes/no)
8. Did you, since you received the last HPV vaccine (about 9 months ago), experience any signs or symptoms that you did not report during the previous questionnaire? If yes, please describe

**Appendix II: Supplemental Methods, Sample Size Calculations**

The sample size calculation was informed by data from the PASSYON study^2^, specifically using data from MSM aged 16-24 years who enrolled at the Amsterdam SHC in this study. Of those, 13.9% tested positive for anal HPV-16 infection (baseline prevalence π_1_), 13.9% tested negative for anal HPV-16 infection but positive for HPV-16 specific serum antibodies (assumed proportion with natural immunity; Imm), and 72.2% tested DNA- and seronegative for HPV-16. In addition, 11.3% tested positive for anal HPV-18 infection, 11.9% tested negative for anal HPV-18 infection but positive for HPV-18 specific serum antibodies, and 76.8% tested DNA- and seronegative for HPV-18. For prevalent HPV-16 and HPV-18 infections, i.e. those who test DNA-positive for HPV-16 or HPV-18 at baseline, we assume a 2-year clearance probability of 65% and 70%, respectively, as previously estimated in HIV-negative MSM in Amsterdam.^3^ We assume that, in the absence of vaccination, all clearing infections in this group would be replaced by incident infections (Inc), in line with the notion of non-decreasing HPV prevalence by age in this population.

The HPV-16 infection prevalence at month 24 (π_2_) is a result of the prevalence at baseline (π_1_ = 0.139) minus the clearance during the 24-month follow-up (κ = 0.65), plus incidence during 24-month follow-up (see Figure S1). Hence, this is calculated as π_2_ = π_1_ ∙ (1 –κ) + (1 – π_1_) ∙ (1 – ϕ) ∙ Inc ∙ (1 – θ) = 0.062, with ϕ = Imm/(1 – π_1_) denoting the proportion with natural immunity among those without baseline HPV-16 infection (**Figure 2 & Supplementary Figure S1**).

The HPV-positivity at month 24 amounts to 0.062 which needs to be tested against the reference value under H_0_ of 0.139. At 5% significance, the required sample size to achieve 80% statistical power (i.e. type II error probability of 20%) would then be 264 vaccinated and 264 unvaccinated for HPV-16. For HPV-18, conducting a similar line of reasoning, the required sample size would be 284 vaccinated and 284 unvaccinated. To retain power when estimating VE in analyses with adjustment for confounders, we would require approximately 300 vaccinated and 300 unvaccinated MSM. More vaccinated MSM need to be enrolled at month 0 to cover expected loss to follow-up. We conservatively estimated this loss to be 30%, so a total of 430 participants need to be included for Group 1.

**References**

1. Palefsky JM, Giuliano AR, Goldstone S, et al. HPV vaccine against anal HPV infection and anal intraepithelial neoplasia. *N Engl J Med* 2011;365(17):1576-85. doi: 10.1056/NEJMoa1010971 [published Online First: 2011/10/28]

2. Mooij SH, van Santen DK, Geskus RB, et al. The effect of HIV infection on anal and penile human papillomavirus incidence and clearance: a cohort study among MSM. *AIDS* 2016;30(1):121-32. doi: 10.1097/qad.0000000000000909

3. Woestenberg PJ, van Benthem BHB, Bogaards JA, et al. HPV infections among young MSM visiting sexual health centers in the Netherlands: Opportunities for targeted HPV vaccination. *Vaccine* 2020;38(17):3321-29. doi: <https://doi.org/10.1016/j.vaccine.2020.03.002>
