# Supplementary material for: Human papillomavirus (HPV) vaccine effectiveness against anal HPV-16 and HPV-18 infections among young men who have sex with men visiting the sexual health center of Amsterdam (HPV4M): study protocol of an observational study
Source: BMJ Open. 2025 Aug 26;15(8):e101634. doi: 10.1136/bmjopen-2025-101634 (PMC12410653; doi:10.1136/bmjopen-2025-101634)
Supplement: Supplementary file 2 [file bmjopen-2025-101634.reviewer_comments.pdf]

PEER REVIEW HISTORY

BMJ Open publishes all reviews undertaken for accepted manuscripts. Reviewers are asked to complete a checklist review form and are provided with free text boxes to elaborate on their assessment. These free text comments are reproduced below.

ARTICLE DETAILS

Title (Provisional)

Human papillomavirus (HPV) vaccine effectiveness against anal HPV-16 and HPV-18 infections among young men who have sex with men visiting the Sexual Health Center of Amsterdam (HPV4M): study protocol of an observational study

Authors

van Veelen, Sarah; Alberts, Catharina; Hoornenborg, Elske; Bruisten, Sylvia M; Vermeij, Koenraad; van der Klis, Fiona; Noordpool, Luann; Bogaards, Johannes A.; Schim van der Loeff, Maarten Franciscus

---

VERSION 1 - REVIEW

**Reviewer**	**1**
**Name**	**Garcia-Iglesias, Jaime**
**Affiliation**	**The University of Edinburgh**
**Date**	**18-Apr-2025**
**COI**	**None**

I recommend publication.

**Reviewer**	**2**
**Name**	**Harfouch, Omar**
**Affiliation**	**University of Maryland**
**Date**	**25-Jun-2025**
**COI**	**None**

Reviewer Comments:

The protocol presents a well-structured and thoughtful approach to an important research question. It commendably outlines both its strengths and limitations, and it employs a novel methodology for analyzing observational data without relying on a randomized controlled trial (RCT).

However, several clarifications are needed to strengthen the protocol:

1. Definition of MSM:

Please clarify how MSM were defined in the study. Specifically:

• Were all participants cisgender men who have sex with other cisgender men?

• Was there a specific time frame used to define recent sexual activity (e.g., within the past year)?

• Were partners of transgender women included in this definition?

2. Composition of Group 2:

It is unclear whether Group 2 includes MSM who were offered the vaccine but declined. This group is mentioned in the discussion as potentially introducing confounding due to differing attitudes toward vaccination. Please clarify whether this population was included or excluded from the analysis.

3. Consideration of Immunocompromising Conditions:

Has the protocol accounted for immunocompromising conditions or the use of immunosuppressive medications? Additionally, were CD4 count thresholds considered for participants living with HIV? These factors could significantly influence vaccine effectiveness and should be addressed.

4. Potential for Confounding Bias:

The vaccinated cohort may represent individuals with higher health literacy and greater awareness of HPV-related risks. This could introduce confounding bias, and it would be helpful to see this acknowledged and discussed in the limitations section.

5. Handling of Missed Doses:

What is the planned approach for participants who miss scheduled vaccine doses? Will there be a protocol for adjusting the vaccination schedule, and how will this be accounted for in the analysis?

VERSION 1 - AUTHOR RESPONSE

**Responses to Reviewer Comments**

Reviewer: 1
Dr. Jaime Garcia-Iglesias, The University of Edinburgh

Comments to the Author:
I recommend publication.

Response: thank you for this clear and positive recommendation.

Reviewer: 2
Dr. Omar Harfouch, University of Maryland

1. Definition of MSM:

Please clarify how MSM were defined in the study. Specifically:

• Were all participants cisgender men who have sex with other cisgender men?
• Was there a specific time frame used to define recent sexual activity (e.g., within the past

year)?

- Were partners of transgender women included in this definition?

Response: Thank you for indicating this. We now provide a clearer definition of MSM. We have revised the "Study population" subsection of the Methods and analysis section to explicitly define the inclusion criteria regarding sex assigned at birth, gender identity, and recent sexual activity. The updated text clarifies the timeframe used and the inclusion of gender-diverse individuals.

**Original text (p16)**	**Revised text (p8)**
Participants are recruited at the SHC of Amsterdam in two phases. During the first recruitment phase, MSM born between 1st of January 1996 and 31st of December 2003 (aged 19-26 years at the start of the catch-up campaign) attending the SHC and accepting HPV vaccination, were invited to participate (Group 1). See Table 1 for eligibility criteria.	Participants are recruited at the SHC of Amsterdam in two phases. During the first recruitment phase, MSM born between 1st of January 1996 and 31st of December 2003 (aged 19-26 years at the start of the catch-up campaign) attending the SHC and accepting HPV vaccination, were invited to participate (Group 1). MSM were defined as individuals who were assigned male sex at birth, had not undergone genital gender- affirming surgery, regardless of their current gender identity, and who reported having had sex with partners with a penis in the past 6 months.<br>See Table 1 for remaining eligibility criteria.

2. Composition of Group 2:

It is unclear whether Group 2 includes MSM who were offered the vaccine but declined. This group is mentioned in the discussion as potentially introducing confounding due to differing attitudes toward vaccination. Please clarify whether this population was included or excluded from the analysis.

Response: Thank you for this comment. We agree that it is important to specify whether Group 2 consists solely of individuals who missed the opportunity to get vaccinated or also includes those who actively decline vaccination. To clarify, we have revised the text to provide additional context about the timing of recruitment relative to the national catch-up campaign and to describe the composition of Group 2 more precisely.

**Original text**	**Revised text (p8)**
Group 1 will be followed-up until 24 months after their first vaccination. In the calendar period that Group 1 men return for their 24-month visits, an unvaccinated age-matched control population will be enrolled (Group 2) (see **Figure 1** for schematic representation). Group 2 participants are recruited from the same birth cohorts as Group 1 participants, enabling direct comparison of the prevalence of anal HPV-16 and HPV-18 infections between the vaccinated (Group 1) and the unvaccinated (Group 2) participants.	Group 1 will be followed-up until 24 months after their first vaccination. In the calendar period that Group 1 men return for their 24-month visits, an unvaccinated age-matched control population will be enrolled (Group 2) (see **Figure 1** for schematic representation). Group 2 participants are recruited from the same birth cohorts as Group 1 participants, enabling direct comparison of the prevalence of anal HPV-16 and HPV-18 infections between the vaccinated (Group 1) and the unvaccinated (Group 2) participants. During the recruitment period for Group 1, HPV vaccination was available free of charge through the national catch-up campaign for all individuals up to the age of 26. Group 2 is recruited after the campaign was concluded, and free vaccination was no longer available. Group 2 participants were asked about their reasons for not having received the HPV vaccine. Participants were eligible for participation in Group 2 of the study, if they would be willing to get vaccinated if offered the vaccine at enrolment.

3. Consideration of Immunocompromising Conditions:

Has the protocol accounted for immunocompromising conditions or the use of immunosuppressive medications? Additionally, were CD4 count thresholds considered for participants living with HIV? These factors could significantly influence vaccine effectiveness and should be addressed.

Response: People with immunocompromising conditions or the use of immunosuppressive medications were not excluded from participation. Conditions and medications were registered, and these participants were offered three vaccine doses, at day 0, month 1, and month 6, in line with the national guidelines. Such conditions or medications may indeed affect vaccine effectiveness. Therefore, we have now added a sensitivity analysis excluding people with immunocompromising conditions, the use of immunosuppressive medications, or HIV infection.

For participants with HIV we collect data on CD4 counts and viral load; people with HIV attending the SHC are generally on antiretroviral treatment, have high CD4 counts and have suppressed viral loads. We added this additional information as follows:

**Original text**	**Revised text (p8)**
In the Netherlands, the bivalent HPV vaccine is routinely offered to preadolescent girls since 2009 and vaccination was extended to boys in 2022. In 2023, a catch-up campaign was launched to offer the bivalent vaccine to all unvaccinated individuals up to the age of 26. The vaccination programme uses a 2-dose schedule in accordance with the Dutch national vaccination programme31, i.e. vaccine is given at month 0 and at month 6. People with HIV or other causes of immunosuppression are given an extra dose at month 1.<br><br>Of note, vaccination leads to seroconversion and very high antibody concentrations, also in the large majority in people with HIV.[32-36] Therefore we did not exclude MSM with HIV from participation. The expected number of MSM with HIV in this age group is low (<5% of eligible men), so stratified analyses by HIV status will not be feasible.	In the Netherlands, the bivalent HPV vaccine is routinely offered to preadolescent girls since 2009 and vaccination was extended to boys in 2022. In 2023, a catch-up campaign was launched to offer the bivalent vaccine to all unvaccinated individuals up to the age of 26. The vaccination programme uses a 2-dose schedule in accordance with the Dutch national vaccination programme31, i.e. vaccine is given at month 0 and at month 6. People with HIV or other causes of immunosuppression are given an extra dose at month 1.<br><br>Of note, vaccination leads to seroconversion and very high antibody concentrations, also in the large majority in people with HIV.[32-36] Therefore we did not exclude MSM with HIV from participation. For participants with HIV, CD4 cell counts and HIV viral load data are collected.<br>The expected number of MSM with HIV in this age group is low (<5% of eligible men), so stratified analyses by HIV status will not be feasible.<br>In the analysis we will conduct a sensitivity analysis excluding people with immunocompromising conditions, the use of immunosuppressive medications, or HIV infection

4. Potential for Confounding Bias:

The vaccinated cohort may represent individuals with higher health literacy and greater awareness of HPV-related risks. This could introduce confounding bias, and it would be helpful to see this acknowledged and discussed in the limitations section.

Response: Thank you for this important point. In principle Group 2 participants are included on the hypothetical basis that they agree to be vaccinated. Nevertheless, the fact they have not yet been vaccinated whereas Group 1 participants have been, may suggest differences in health literacy and awareness of HPV-related risks. Our main multivariable analysis will adjust for possible differences between Group 1 and 2 participants. We agree with the Reviewer's suggestion to highlight the possibility of remaining confounding in the Discussion.

**Original text**	**Revised text (p18-19)**
By recruiting all participants at the same SHC using the same approach and protocol, we increase comparability between the vaccinated and unvaccinated groups in the absence of randomisation. Furthermore, we anticipated that recruiting an unvaccinated control group during the campaign could introduce confounding, as those who choose to get vaccinated may differ systematically from those who do not. To address this, the unvaccinated group will be recruited at a later stage and will consist of men who missed the opportunity for HPV vaccination rather than those who explicitly declined it.	By recruiting all participants at the same SHC using the same approach and protocol, we increase comparability between the vaccinated and unvaccinated groups in the absence of randomisation. ~~Furthermore,~~ We anticipated that recruiting an unvaccinated control group during the campaign could introduce confounding, as those who choose to get vaccinated during the campaign may differ systematically from those who do not, for example, in terms of health literacy and awareness of HPV related risks. To address this, the unvaccinated group will be recruited at a later stage (when the HPV catch-up campaign is concluded) and will consist of men who missed the opportunity for HPV vaccination rather than those who explicitly declined it. Attendees were eligible for participation in Group 2 of the study, if they would be willing to get vaccinated if offered the vaccine at enrolment. Furthermore, the analysis will adjust for variables on which the two groups differ. Nevertheless, residual confounding may occur.

5. Handling of Missed Doses:

What is the planned approach for participants who miss scheduled vaccine doses? Will there be a protocol for adjusting the vaccination schedule, and how will this be accounted for in the analysis?

Response: Thank you for raising this issue. We have updated the *Procedures and Interventions* and *Statistical Analysis* sections to clarify our approach to missed doses, rescheduling, and the impact on per-protocol analyses.

**Original text**	**Revised text**
P. 10 (procedures and interventions):<br>At month 6, participants receive their last vaccine dose. No samples for testing are collected at month 1 (for participants with HIV) nor month 6. At month 15, participants receive a short online questionnaire about sexual behaviour and potential long-term adverse events (AE). During the 24-month visit, participants are asked to complete the same online questionnaire and provide the same anal, penile, oral and blood samples as during the initial appointment. Study participation is completed after this visit.<br><br>P14 (statistical analyses):<br>We will provide both intention-to-treat (ITT) and per-protocol (PP) estimates of VE. In the ITT analysis, all individuals who are recruited into Group 1 and return for the month 24 visit will be included. In the PP analysis, we will only consider Group 1 participants who received all vaccine doses in a timely manner.	P. 10 (procedures and interventions):<br>At month 6, participants receive their last vaccine dose. Participants who missed a scheduled dose are contacted by phone and mail to reschedule the appointment as soon as possible. No samples for testing are collected at month 1 (for participants with HIV) nor at month 6. At month 15, participants ~~receive~~ complete a short online questionnaire about sexual behaviour and ~~potential long-term~~ adverse events (AE). During the 24-month visit, participants are asked to complete the same online questionnaire and provide the same anal, penile, oral and blood samples as during the initial appointment. Participants who have missed their 6-month (or 1-month, in case of immunocompromised individuals and people with HIV) vaccine dose, are offered vaccination at the 24-month study visit.<br>Study participation is completed after this visit.<br><br>P14 (statistical analyses):<br>We will provide both intention-to-treat (ITT) and per-protocol (PP) estimates of VE. In the ITT analysis, all individuals who are recruited into Group 1 and return for the month 24 visit will be included. In the PP analysis, we will only consider Group 1 participants who received all vaccine doses in the recommended intervals. Recommended intervals are 5-12 months after the first dose for the 6-month dose, and 3 to 6 weeks after dose 1 for the 1-month dose in immunocompromised individuals.[1,2]

**Additional minor changes**

In addition to the revisions made in response to your comments, we have made several minor textual changes to improve clarity and consistency, listed below. Furthermore, we added a figure legend on the last page of the manuscript, added the guarantors to the contributor statement and added a novel competing interest for one of the co-authors (JB).

P.7 Section *Methods and analysis*, subsection *Project status*.

Original text	Revised text
In February 2025, recruitment of Group 2 and 24-month visits of Group 1 are commenced	In February 2025, recruitment of Group 2 and 24-month visits of Group 1 ~~are~~ commenced

P.7 Section *Methods and analysis*, subsection *Objectives*. We revised the description of the setting to specify the Sexual Health Center of Amsterdam to improve clarity and consistency with the manuscript title

Original text	Revised text
Here we describe an observational study design to estimate the HPV vaccine effectiveness in a real-world setting for MSM	Here we describe ~~an~~ the observational study design to estimate the HPV vaccine effectiveness ~~in a real-world setting~~ for MSM visiting the SHC of Amsterdam

P. 10 Section *Methods and analysis,* subsection *Procedures and interventions.* We revised this section slightly, as according to the HPV4M protocol, all reported adverse events within scope are assessed, not only long-term adverse events

Original text	Revised text
No samples for testing are collected at month 1 (for participants with HIV) nor month 6. At month 15, participants receive a short online questionnaire about sexual behaviour and potential long-term adverse events (AE).	No samples for testing are collected at month 1 (for participants with HIV) nor ~~at~~ month 6. At month 15, participants ~~receive~~ complete a short online questionnaire about sexual behaviour and ~~potential long-term~~ adverse events (AE).

p. 13 Section *Methods and Analysis,* subsection *Sample size calculation*

Original text	Revised text
see Supplementary Table S2 and Figure S12, and Appendix II for formula and calculations	see Supplementary Table S~~2~~1 and Figure S12, and Appendix II for formula and calculations

P. 15 Section *Methods and Analysis,* subsection *Patient and Public involvement*.

Original text	Revised text
Patient and/or public were involved in the design plan of this research	Patient and/or public were involved in the design ~~plan~~ of this research

P.26 Table 1. We changed 'Clinic' to 'Center' to ensure consistency in the whole manuscript

Original text	Revised text
SHC = Sexual Health Clinic	SHC = Sexual Health ~~Clinic~~ Center

References

1. Centers for Disease Control and Prevention. HPV vaccination: Recommendations of the ACIP. (2021).
2. Rijksinstituut voor Volksgezondheid en Milieu. Tijdstip van vaccinaties (Rijksvaccinatieprogramma) [in Dutch]. (2025).

VERSION 2 - REVIEW

**Reviewer**	**2**
**Name**	**Harfouch, Omar**
**Affiliation**	**University of Maryland**
**Date**	**07-Aug-2025**
**COI**	

I recommend publication
